# The m6A methyltransferase METTL3 drives thyroid cancer progression and lymph node metastasis by targeting LINC00894

**DOI:** 10.1186/s12935-024-03240-5

**Published:** 2024-01-30

**Authors:** Xiang Zhou, Lisha Chang, Qiaoqiao Liang, Rongjie Zhao, Yong Xiao, Zheng Xu, Leitao Yu

**Affiliations:** 1 Head and neck surgery, The Second Affiliated Hospital of Nanchang University, Nanchang City, Jiangxi Province People’s Republic of China; 2 Oncology department, The Second Affiliated Hospital of Nanjing Medical University, Nanjing City, People’s Republic of China; 3 State Key Laboratory of Reproductive Regulation and Breeding of Grassland Livestock, School of Life Sciences, Inner Mongolia University, Hohhot, People’s Republic of China

**Keywords:** Papillary thyroid carcinoma, METTL3, LINC00894, Hippo signalling pathway, m6A modification

## Abstract

**Background:**

Long noncoding RNAs (lncRNAs) are significant contributors to various human malignancies. The aberrant expression of lncRNA LINC00894 has been reported in various human malignancies. We aimed to illustrate the role of LINC00894 and its underlying mechanism in the development of papillary thyroid carcinoma (PTC).

**Methods:**

We performed bioinformatics analysis of differentially expressed RNAs from TCGA and GEO datasets and selected the target lncRNA LINC00894. SRAMP analysis revealed abundant M6A modification sites in LINC00894. Further analysis of StarBase, GEPIA, and TCGA datasets was performed to identify the related differentially expressed genes METTL3. Colony formation and CCK-8 assays confirmed the relationship between LINC00894, METTL3, and the proliferative capacity of PTC cells. The analysis of AnnoLnc2, Starbase datasets, and meRIP-PCR and qRT‒PCR experiments confirmed the influence of METTL3-mediated m6A modification on LINC00894. The study employed KEGG enrichment analysis as well as Western blotting to investigate the impact of LINC00894 on the expression of proteins related to the Hippo signalling pathway.

**Results:**

LINC00894 downregulation was detected in PTC tissues and cells and was even further downregulated in PTC with lymphatic metastasis. LINC00894 inhibits the lymphangiogenesis of vascular endothelial cells and the proliferation of cancer cells. METTL3 enhances PTC progression by upregulating LINC00894 by enhancing LINC00894 mRNA stability through the m6A-YTHDC2-dependent pathway. LINC00894 may inhibit PTC malignant phenotypes through the Hippo signalling pathway.

**Conclusion:**

The METTL3-YTHDC2 axis stabilizes LINC00894 mRNA in an m6A-dependent manner and subsequently inhibits tumour malignancy through the Hippo signalling pathway.

## Introduction

Recently, the incidence of thyroid cancer has increased significantly, and its incidence is increasing faster than that of many other malignancies [1, 2]. In China, there are over ninety thousand new thyroid cancer cases every year [3]. Differentiated thyroid carcinoma (DTC) accounts for approximately 90% of all thyroid cancers, and papillary thyroid carcinoma (PTC) is the most prevalent subtype [4]. There is a higher likelihood of lymph node metastasis for early PTC than for other types [5], and because the symptoms of PTC are nonspecific, lymph node metastasis often occurs before diagnosis and is the main reason underlying the progression and recurrence of PTC; moreover, this event consider worsens patients’ quality of life and prognosis [6]. Therefore, early identification of PTC lymph node metastasis can help reduce the recurrence rate and improve patient prognosis.

Long noncoding RNAs (lncRNAs), noncoding RNAs (ncRNAs) ranging in length from 200 to 100,000 nucleotides and are usually located in the nucleus and cytoplasm [7]. LncRNAs may play a key role in cancer development by regulating chromatin remodelling [8], transcription [9], RNA degradation [10], translation regulation [11], and other pathways. Currently, multiple studies have found that lncRNAs have a significant effect on tumour progression and lymph node metastasis [12, 13], including PTC [14, 15]. LINC00894 overexpression was found to exhibit a close association with the proliferative and invasive abilities of kidney and lung cancers [16, 17]. In breast cancer, LINC00894 enhances breast cancer proliferative and invasive capabilities via competitive binding with miR-429 [18]. These studies suggest that dysregulation of LINC00894 contributes to tumour progression, and its function varies with tumour type. Nevertheless, the precise mechanism underlying the involvement of LINC00894 in PTC progression and lymph node metastasis remains unclear.

M6A modification, the most common RNA modification, is catalysed by a multicomponent methyltransferase complex, and the methyltransferase METTL3 is involved in the formation of m6A as its catalytic core [19, 20]. METTL3 was shown to be involved in tumour progression, such as promoting the progression of colon cancer through tumour immunosuppression [21], mediating acetylation to promote the progression of breast cancer [22], and regulating the m6A modification level of mRNA to facilitate the progression of bladder cancer [23]. The promotion of cancer in the majority of tumours is associated with the upregulation of METTL3 expression; however, the mechanism underlying its role is different. Its latent function in PTC and the mechanism by which METTL3 inhibits the aggressive phenotype of PTC are still unclear.

In this study, a comprehensive analysis of public databases and tissue microarrays was conducted to identify lncRNAs with differential expression between normal thyroid tissue, PTC, and PTC with lymph node metastases. The results indicated a significant progressive decrease in LINC00894 expression these three tissue types. According to the study, LINC00894 in thyroid cancer has a close correlation with lymph node metastasis and a poor prognosis. We then identified the biological function of LINC00894, and the results of functional experiments revealed that LINC00894 overexpression caused significant inhibition of cell proliferation and lymphatic metastasis in vitro. Furthermore, we identified LINC00894 as a probable direct target modulated by the METTL3-YTHDC2 axis and confirmed that LINC00894 exerts its biological effect by modulating the Hippo signalling pathway. These findings highlight new molecular mechanisms for thyroid cancer development and new treatment approaches.

## Materials and methods

### Analysis of public databases

Datasets for this study are available in the Gene Expression Omnibus (GEO) database or in the Cancer Genome Atlas (TCGA) database. 30 normal thyroid tissues, 33 PTC tissues without lymphatic metastasis, and 23 PTC tissues with lymphatic metastasis were included in this study from the public dataset. Level-three expression data obtained using RNA sequencing, together with the corresponding clinical data for PTC patients, were obtained from the TCGA dataset, accessible at (https://portal.gdc.com). The GTEx datasets, specifically the V8 version, were acquired via the official GTEx data portal website (https://www.gtexportal.org/home/datasets).

### Cell lines and cell culture

The Nthy-ori3-1 cell line, representing thyroid follicular epithelial cells, and three PTC cell lines (KTC-1, BCPAP, TPC-1) were procured from the Cell Bank of the Chinese Academy of Sciences (Shanghai, China). Additionally, human umbilical vein endothelial cells (HUVECs) were procured from American Type Culture Collection (Manassas, VA, USA). The cell culture was maintained in RPMI-1640 medium (Gibco, Grand Island, NY, USA) that contained 1% penicillin/streptomycin and 10% foetal bovine serum (FBS). Subsequently, the cell lines were subjected to incubation at 37 °C and 5% CO2 [24].

### Cell transfection

The following constructs were procured from RiboBio Co. (Guangzhou, China): pcDNA empty vector, pcDNA-LINC00894, pcDNA-METTL3, pcDNA-YTHDC2, siRNA normal control (SI-NC), and siRNAs targeting LINC00894 (SI-LINC00894). The process of transfection was carried out utilizing either Lipofectamine 2000 (Invitrogen, Carlsbad, CA, USA) or X-tremeGENE transfection reagent (Thermo Fisher Scientific, USA) following the manufacturer’s protocols [25].

### RNA extraction, reverse transcription, and real-time PCR

TRIzol reagent (Invitrogen, Carlsbad, CA, USA) was utilized to isolate total RNA from the cells following the manufacturer’s protocols. Subsequently, 1 µg RNA was subjected to reverse transcription with a Qiagen kit (Qiagen, Valencia, CA, USA) to obtain cDNA. The resulting cDNA was subjected to qRT‒PCR utilizing the SYBR Premix Ex Taq Kit (Takara, Otsu, Japan) on the StepOnePlus™ Real-Time PCR System (Applied Biosystems; Shanghai, China). The 2^−ΔΔCt^ method was utilized to quantify relative expression, with GAPDH serving as the endogenous control [26]. The primer sequences were as follows:

LINC00894: forward: 5′-CCAAATCTGACACACCATAGC-3′;

reverse: 5′-GAACACAGCATGCAGGTAAT-3′.

GAPDH: forward: 5′-GCTGTAGCCAAATCGTTGT-3′,

reverse: 5′-CCAGGTGGTCTCCTCTGA-3′.

METTL3: forward: 5′-AAGCTGCACTTCAGACGAAT-3′,

reverse: 5′-GGAATCACCTCCGACACTC-3′.

YTHDC2: forward: 5′-CAAAACATGCTGTTAGGAGCCT-3′,

reverse: 5′- CCACTTGTCTTGCTCATTTCCC-3′.

### Subcellular RNA fractionation

Nuclear and cytoplasmic RNA was extracted using the NE-PER Nuclear and Cytoplasmic Extraction Kit (Thermo Fisher Scientific, Waltham, MA, USA) according to the manufacturer’s protocol. Briefly, approximately 10 × 10^4^ cells were suspended in 300 µl of fractionation buffer and then centrifuged at 4°C to isolate the cytoplasmic fraction. The nuclear pellet was then fragmented with cell disruption buffer, and the same volume of 2X lysis/conjugation solution as the RNA lysate was added to the nuclear lysis product, which was finally treated with 100% ethanol. qRT‒PCR was performed to detect the relative RNA levels in the nucleus and cytoplasm. U6 was used as a positive nuclear control, and GAPDH was used as a cytoplasmic control [25, 26].

### Fluorescence in situ hybridization

PTC cells were inoculated in 24-well plates, and when the cells grew to a suitable density, the medium was discarded and the cells were washed with PBS. Subsequently, the cells were fixed with 4% PFA paraformaldehyde, washed and added with prehybridization solution at 65 °C for 1 h. Then the hybridization solution containing the probe was added and reacted in the dark at 65 °C in a hybridizer for 48 h. The cells were washed with 4× and 2× SSC, and then subjected to DAPI staining. The slices were observed by fluorescence microscopy after sealed [27]. RNA FISH probes were designed and synthesized by Ribobio (Guangzhou, China).

### Tube formation assay

Precooled Matrigel was added to a 96-well plate and subjected to incubation at 37 °C for 30 min. HUVECs (2 × 10^4^ cells/well) were suspended in 200 µL of conditioned medium and subsequently transferred to the corresponding well and incubated with supernatants from specified KTC-1 and BCPAP cells in 5% CO_2_ at 37 °C for 8–12 h [28]. The images of tube structures were detected under an inverted bright-field microscope at 100× magnification, and photographs were taken. ImageJ software was used to determine the number of meshes.

### Cell counting Kit-8 (CCK-8) assay

Cell viability was assessed through CCK-8 assays [29]. KTC-1 and BCPAP cells were resuspended and subsequently seeded into 96-well plates with a total of five wells allocated to each group. Every well was filled with 2 × 10^3^ cells and 100 µL of medium, which was then subjected to incubation at 37 °C with 5% CO2 for 0, 1, 2, 3, 4, and 5 consecutive days. The 10 µL CCK-8 solution (KeyGEN, Nanjing, China) was pipetted into each well, followed by a 2-hour incubation period. The cellular proliferation rate was measured at 450 nm absorbance through an enzymatic marker.

### 5-Ethynyl-2′-deoxyuridine incorporation assay

Cell proliferation was assessed using the 5-ethynyl-2′-deoxyuridine (EdU) assay kit (RiboBio) according to the manufacturer’s guidelines [26, 29–31]. Tumour cells were inoculated into 96-well plates(2 × 10^4^ cells/well ) 24 h after transfection. After 18–24 h of cell culture, the cells were treated with 50 µM EdU for 2 h at 37 °C, fixed with 4% paraformaldehyde, incubated with 2 mg/mL glycine for 5 min, incubated with PBS containing 0.5% Triton X-100 for 10 min, dipped in 1× Apollo staining solution and finally incubated with 100 ml of 1× Hoechst 33,342 for 30 min. The percentage of EdU-positive cells was detected by fluorescence microscopy.

### Colony formation assay

A colony formation assay was utilized to evaluate the colony formation ability of cells. PTC cells were inoculated into 6-well plates (100 cells/well) and then cultured at 37 °C and 5% CO_2_ for 14 days, and the medium was replaced every 3–5 days on a regular basis. After 14 days, the medium was removed, and the cells were fixed with paraformaldehyde. Afterwards, the cells were subjected to staining with 0.1% crystal violet, followed by a 20-min incubation at room temperature (RT). Colonies were counted and photographed [29].

### MeRIP-qPCR

The Magna meRIP M6A Kit (Millipore, Germany) was utilized to conduct the meRIP assay for the purpose of determining m6A enrichment following the manufacturer’s protocols. The PTC cells were subjected to lysis using complete RIP lysis buffer, following which the total RNA was extracted. The RNA samples were subjected to immunoprecipitation with magnetic beads containing either anti-m6A antibody or anti-mouse IgG. The RNAs that were coprecipitated went through a purification process and were subsequently dissolved in RNAse-free water. qRT‒PCR was used to analyse m6A enrichment of binding RNA targets.

### Western blotting analysis

The PTC cells were subjected to protein extraction through RIPA buffer (Beyotime Biotechnology, Shanghai, China) containing protease and phosphatase inhibitors (Merck, Germany). After separating the proteins using SDS‒PAGE, they were transferred onto a polyvinylidene difluoride (PVDF) membrane. The membrane was then blocked for 60 min with QuickBlock™ Western Blocking Buffer (P0252, Beyotime Biotechnology, Shanghai, China) and subjected to incubation overnight at 4 °C. After five washes with TBST for 5 min, the secondary antibody combined with horseradish peroxidase (HRP) was applied for incubation at RT for one hour, and the protein was detected with BeyoECL Moon reagent (Beyotime Biotechnology, Shanghai, China).

### Statistical analysis

Statistical analysis was conducted by SPSS 19.0 software (IBM, Chicago, USA) and GraphPad Prism 8 (San Diego, USA). The study employed one-way analysis of variance (ANOVA) for determining the significance of differences between multiple groups, while Student’s t test was utilized to evaluate the significance of differences between two groups. The data are reported as the mean ± standard deviation (SD). *p* < 0.05 was considered to indicate a significant difference.

## Results

### Low LINC00894 expression is significantly correlated with a poor prognosis and lymphatic metastasis in thyroid cancer patients

**Fig. 1:**
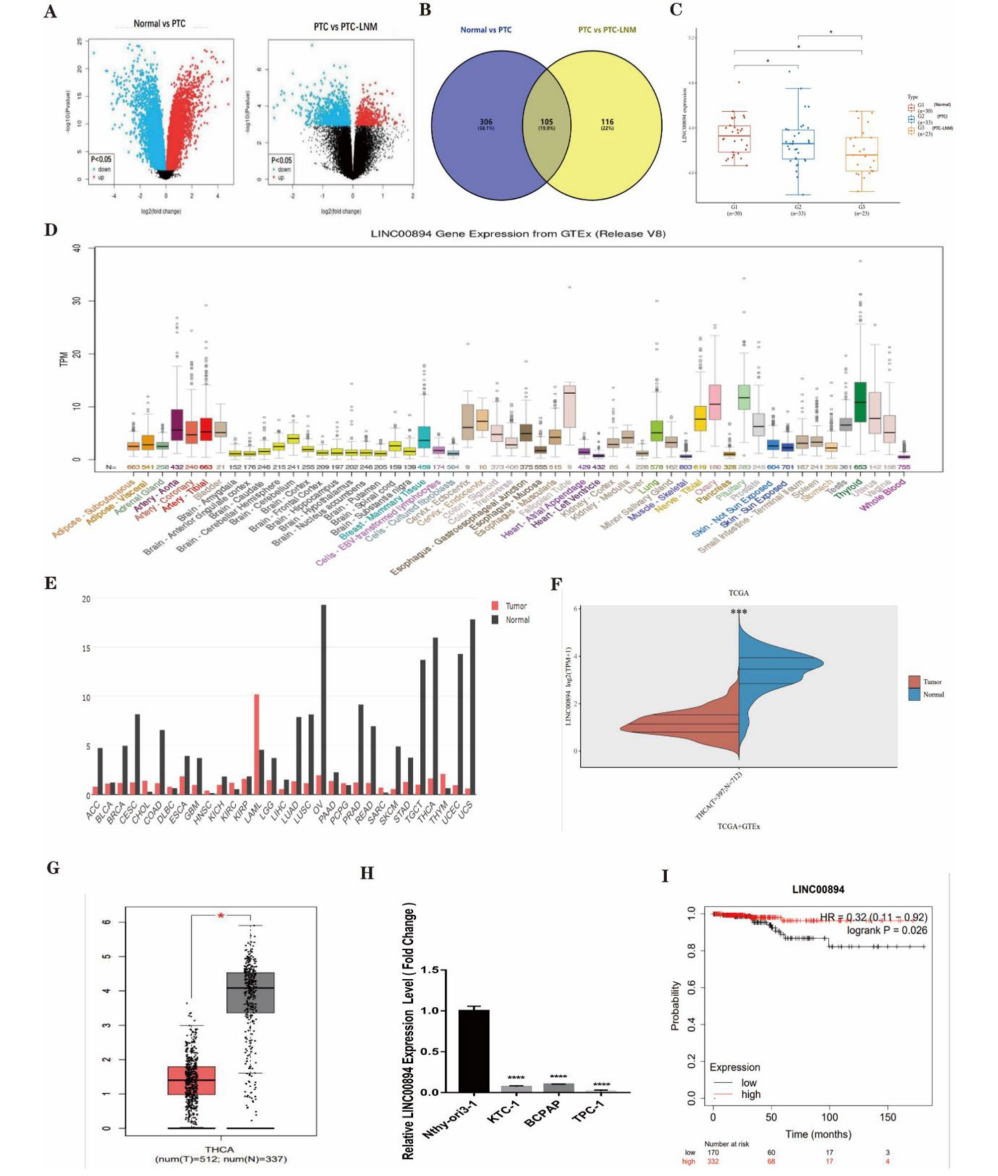
Decreased Expression of LINC00894 in PTC Tissues and Cells. (**A**) Relative LINC00894 expression in normal thyroid tissue, PTC tissue without lymphatic metastasis, and PTC tissue with lymphatic metastasis using GEO (GSE60542). **(B)** Venn diagram showing the overlapping differentially expressed lncRNAs, depending on A1 and A2. **(C)** Assessment of LINC00894 expression in normal thyroid tissue, PTC tissue without lymphatic metastasis, and PTC tissue with lymphatic metastasis (PTC-LNM) using TCGA data. **(D)** Assessment of LINC00894 expression in normal thyroid tissue using the Genotype-Tissue Expression (GTEx) database. **(E)** Assessment of LINC00894 expression in normal and tumour tissues using the Gene Expression Profiling Interactive Analysis (GEPIA) database. **(F)** Assessment of LINC00894 expression in THCA based on TCGA data. **(G)** Assessment of LINC00894 expression in THCA using GEPIA data. **(H)** QRT-PCR analysis of LINC00894 expression levels in PTC cell lines. **(I)** Analysis of the clinical impacts of abnormal LINC00894 expression on PTC patient overall survival using Kaplan‒Meier Plotter. *P < 0.05, **P < 0.01, ***P < 0.001, ****P < 0.0001

To identify lncRNAs strongly associated with PTC, publicly available datasets from the TCGA and GEO were downloaded for bioinformatics analysis (Fig. 1A). Subsequently, lncRNAs differentially expressed in normal thyroid tissue and PTC tissue without lymphatic metastasis, PTC tissue without lymphatic metastasis, and PTC tissue with lymphatic metastasis in the GSE60542 dataset were screened out, and LINC00894 was finally selected as the target lncRNA (Fig. 1B). Further experiments and database analysis demonstrated that the expression of LINC00894 was lower in PTC without lymphatic metastases than in normal thyroid tissue, and LINC00894 was lower in PTC with lymph node metastases than in PTC without lymph node metastases (Fig. 1C-G). According to qRT-PCR data, LINC00894 expression was downregulated in PTC cells compared to that in normal cells (Fig. 1H). Survival analysis suggested that patients with low LINC00894 expression had significantly lower overall survival rates (Fig. 1I). The findings indicate that low LINC00894 expression is related to PTC progression and lymphatic metastasis.

### LINC00894 overexpression inhibits lymphangiogenesis and PTC cell proliferation in vitro

**Fig. 2:**
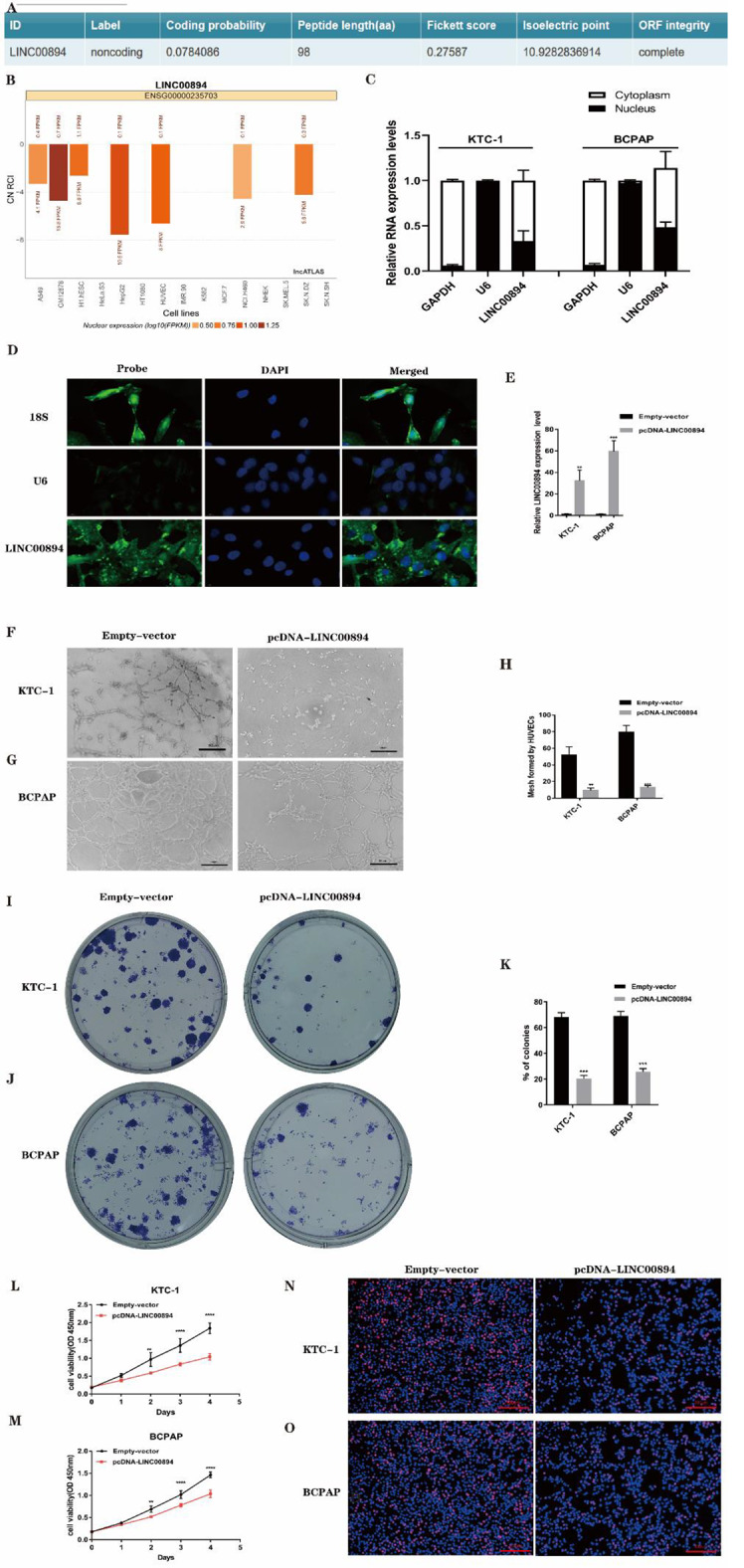
LINC00894 inhibits the lymphangiogenesis of vascular endothelial cells and the proliferation of PTC cells. (**A**) Predicting the coding capability of LINC00894 using the CPC2 database. **(B)** Bioinformatics analyses predicted the subcellular distribution of LINC00894 in various cells. **(C)** Subcellular localization of LINC00894 in KTC-1 and BCPAP cells. The nuclear marker was U6, and the cytosol marker was GAPDH. **(D)** FISH analysis of the location of LINC00894 in the cytoplasm and nuclear fractions of PTC cells. **(E)** Verification of the overexpression efficiency of LINC00894 through qRT‒PCR. **F and H.** Effect of altered LINC00894 expression in PTC cells on HUVEC tube formation. **I and K.** For LINC00894-overexpressing cells, a colony formation assay was used to evaluate PTC cell proliferation. **L and M.** For LINC00894-overexpressing cells, the CCK-8 assay was used to evaluate PTC cell viability. **N and O.** For LINC00894-overexpressing cells, the EdU assay was used to evaluate cell proliferations. **P < 0.01, ***P < 0.001, ****P < 0.0001

To analyse the impact of LINC00894 on the functional phenotype of PTC cells, we first predicted the low coding potential of LINC00894 using CPC2 (http://cpc2.gao-lab.org/index.php) (Fig. 2A). Bioinformatics was also applied to predict the cellular distribution of LINC00894 in various cells, and it was found to be mainly located in the cytoplasm (Fig. 2B). Subsequently, subcellular fractionation and FISH assays were performed in PTC cells. Our results showed that it was more highly expressed in the cytoplasm than in the nucleus (Fig. 2C-D), consistent with the predicted results. Subsequently, the LINC00894 overexpression plasmid was transfected into KTC-1 and BCPAP cells, and the overexpression efficiency of LINC00894 was verified (Fig. 2E). We found that the medium from LINC00894-overexpressing PTC cells inhibited mesh formation by cells (Fig. 2F-H). Colony formation, EdU and CCK-8 assays confirmed that LINC00894 overexpression significantly inhibited PTC cell proliferation (Fig. 2I-O).

### METTL3 expression is closely correlated with LINC00894 expression in PTC

**Fig. 3:**
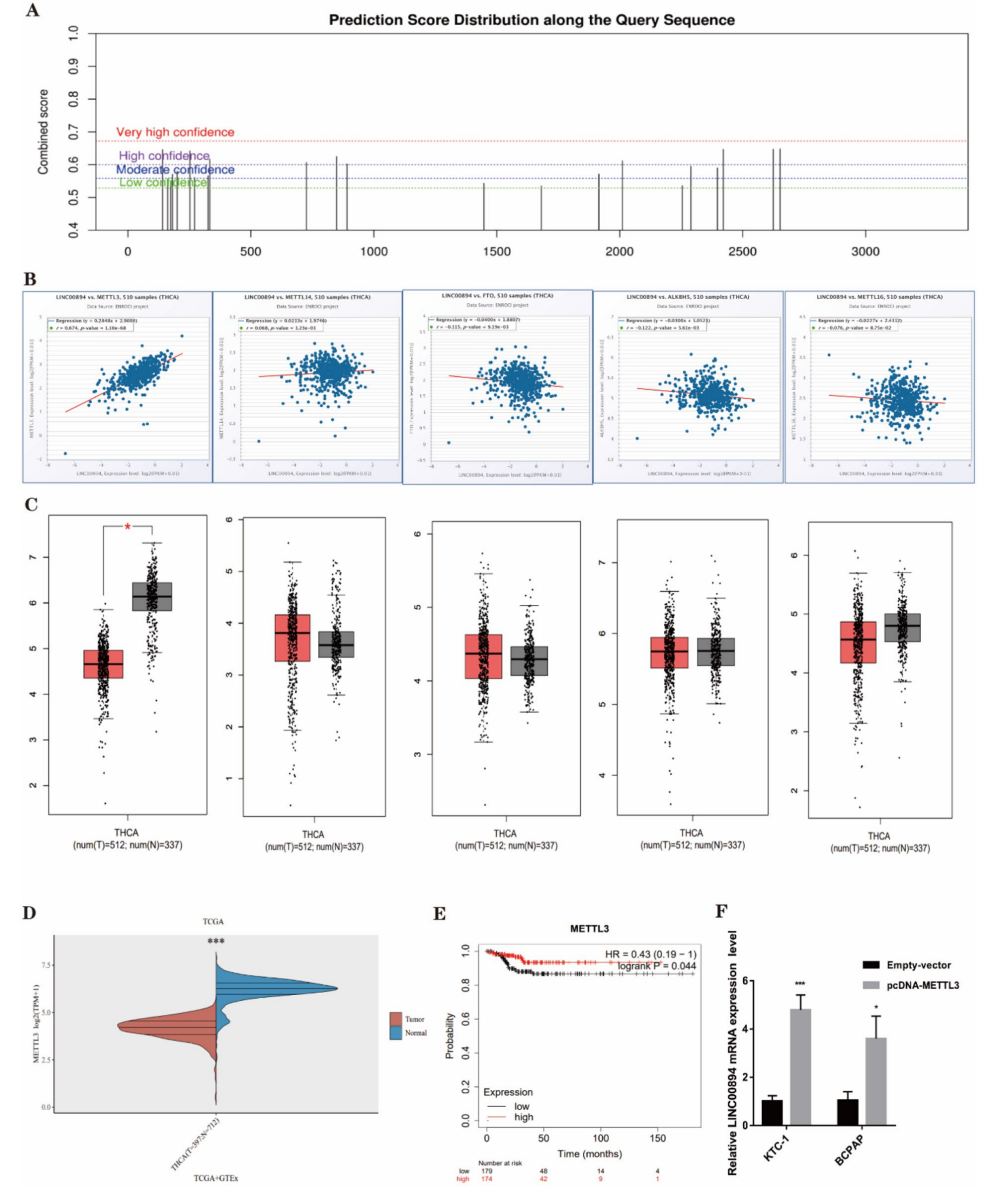
METTL3 is closely associated with the expression of LINC00894 in PTC. (**A**) Prediction of m6A sites of LINC00894 using the SRAMP database. **(B)** Analysis of the correlation between methylases or demethylases and LINC00894 using the starBase database. **(C)** Assessment of methylase or demethylase expression in THCA using GEPIA data. **(D)** Assessment of METTL3 expression in THCA based on TCGA data. **(E)** Assessment of the clinical impacts of abnormal METTL3 expression on PTC patient overall survival using Kaplan‒Meier Plotter. **(F)** Detection of LINC00894 expression following METTL3 overexpression using qRT‒PCR. *P < 0.05, **P < 0.01, ***P < 0.001, ****P < 0.0001

Given the significant impact of m6A modification on gene expression regulation in malignancies, we conducted SRAMP analysis (http://www.cuilab.cn/sramp/), and the results revealed that a large number of m6A modification sites exist in LINC00894 (Fig. 3A). Since m6A modifications are added or removed by a series of methylases or demethylases, such as METTL3, METTL14, METTL16, ALKBH5, FTO, and others [32, 33], to identify the specific genes that lead to changes in m6A modification, we analysed the correlation between related genes and LINC00894 through the StarBase database (https://starbase.sysu.edu.cn/) (Fig. 3B). Subsequently, the expression of relevant enzymes in PTC tissues and normal thyroid tissues was analysed using the GEPIA database (http://gepia.cancer-pku.cn/); the results revealed that METTL3 was significantly downregulated in PTC tissues vs. normal thyroid tissues, and there was no significant difference for other enzymes (Fig. 3C). We further demonstrated that METTL3 was downregulated in PTC through the TCGA database (Fig. 3D). Survival analysis suggested that patients with low levels of METTL3 had significantly lower overall survival rates (Fig. 3E). Subsequently, METTL3 overexpression was found to promote LINC00894 expression in PTC cells (Fig. 3F). The abovementioned results indicate that METTL3 is significantly downregulated in PTC and may influence the expression of LINC00894 through m6A modification.

### METTL3 enhances LINC00894 mRNA stability through the M6A-YTHDC2-dependent pathway

**Fig. 4:**
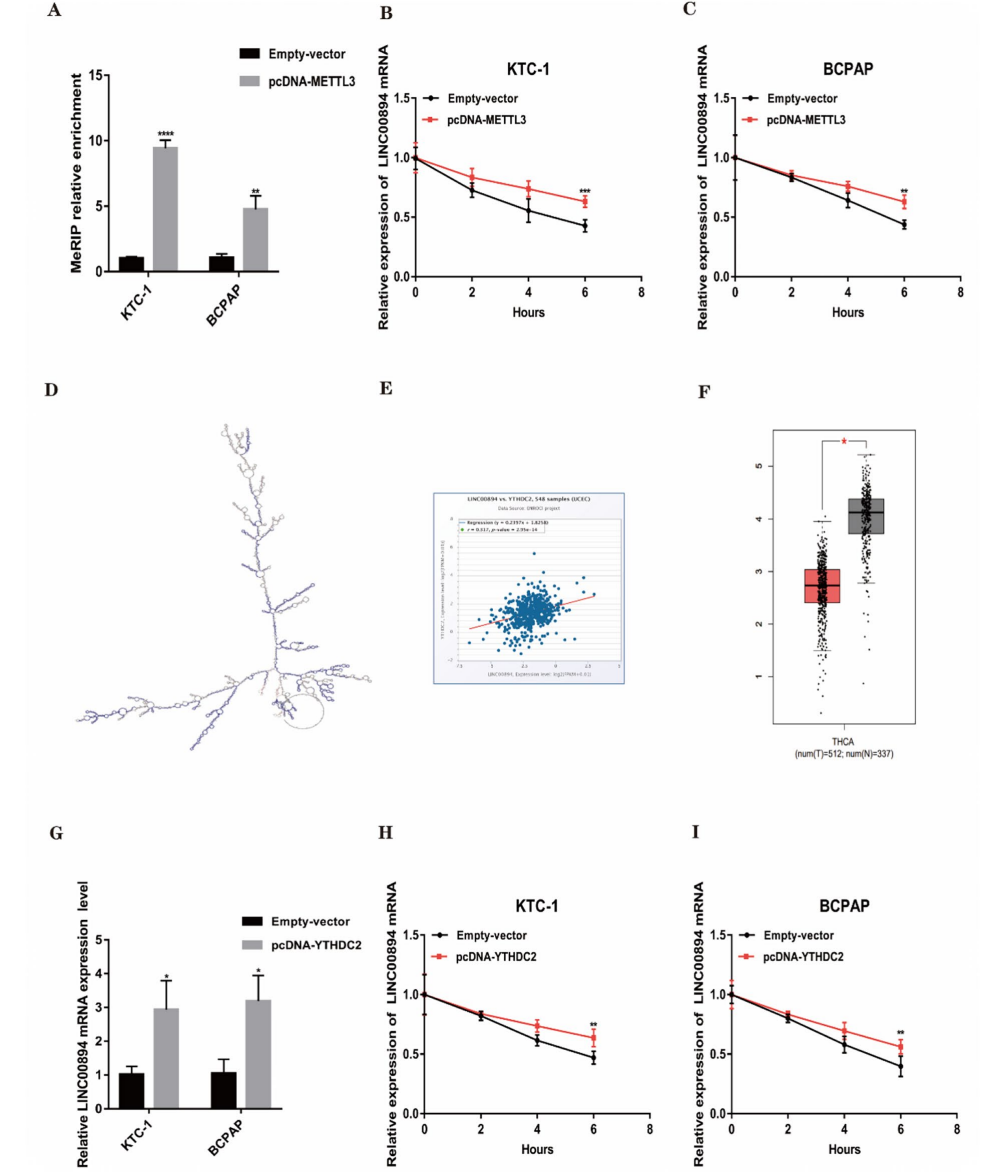
METTL3 enhances LINC00894 mRNA stability through the M6A-YTHDC2-dependent pathway. **A**. Enrichment of METTL3-mediated LINC00894 m6A modification using meRIP-qPCR. **B-C.** Detection of LINC00894 expression in actinomycin D-treated PTC cells overexpressing METTL3 using qRT‒PCR. **D.** Displaying the binding site of YTHDC2 and LINC00894 using the AnnoLnc2 database. **E.** Analysis of the correlation between YTHDC2 and LINC00894 using the starBase database. **F.** Detection of LINC00894 expression using qRT‒PCR following YTHDC2 overexpression. **H-I.** Detection of LINC00894 expression levels in actinomycin D-treated PTC cells overexpressing YTHDC2 using qRT‒PCR. *P < 0.05, **P < 0.01, ***P < 0.001, ****P < 0.0001

To validate the potential impact of METTL3-mediated m6A modification on LINC00894, we conducted meRIP-PCR analysis, which indicated that the m6A level of LINC00894 increased significantly after METTL3 was upregulated (Fig. 4A). As expected, METTL3 overexpression enhanced the stability of LINC00894 mRNA (Fig. 4B-C). Although m6A modification is performed by RNA methyltransferases, m6A-modified RNAs can selectively bind to different m6A readers, resulting in different modifications [34]. We used annonlnc (http://annolnc.gao-lab.org/index.php) to find that LINC00894 has high-affinity binding sites for YTHDC2 (Fig. 4D). Then, we further demonstrated a positive association between YTHDC2 and LINC00894 levels in THCA and confirmed that YTHDC2 was significantly downregulated in PTC tissues (Fig. 4E-F). More importantly, YTHDC2 overexpression was found to elevate LINC00894 expression in PTC cells (Fig. 4G). Similarly, LINC00894 mRNA stability was enhanced when YTHDC2 was overexpressed (Fig. 4H-I). In summary, these findings suggest that METTL3 enhances LINC00894 mRNA stability through the m6A-YTHDC2-dependent pathway.

### Reversal of the tumour-suppressive effects of METTL3 by silencing LINC00894

**Fig. 5:**
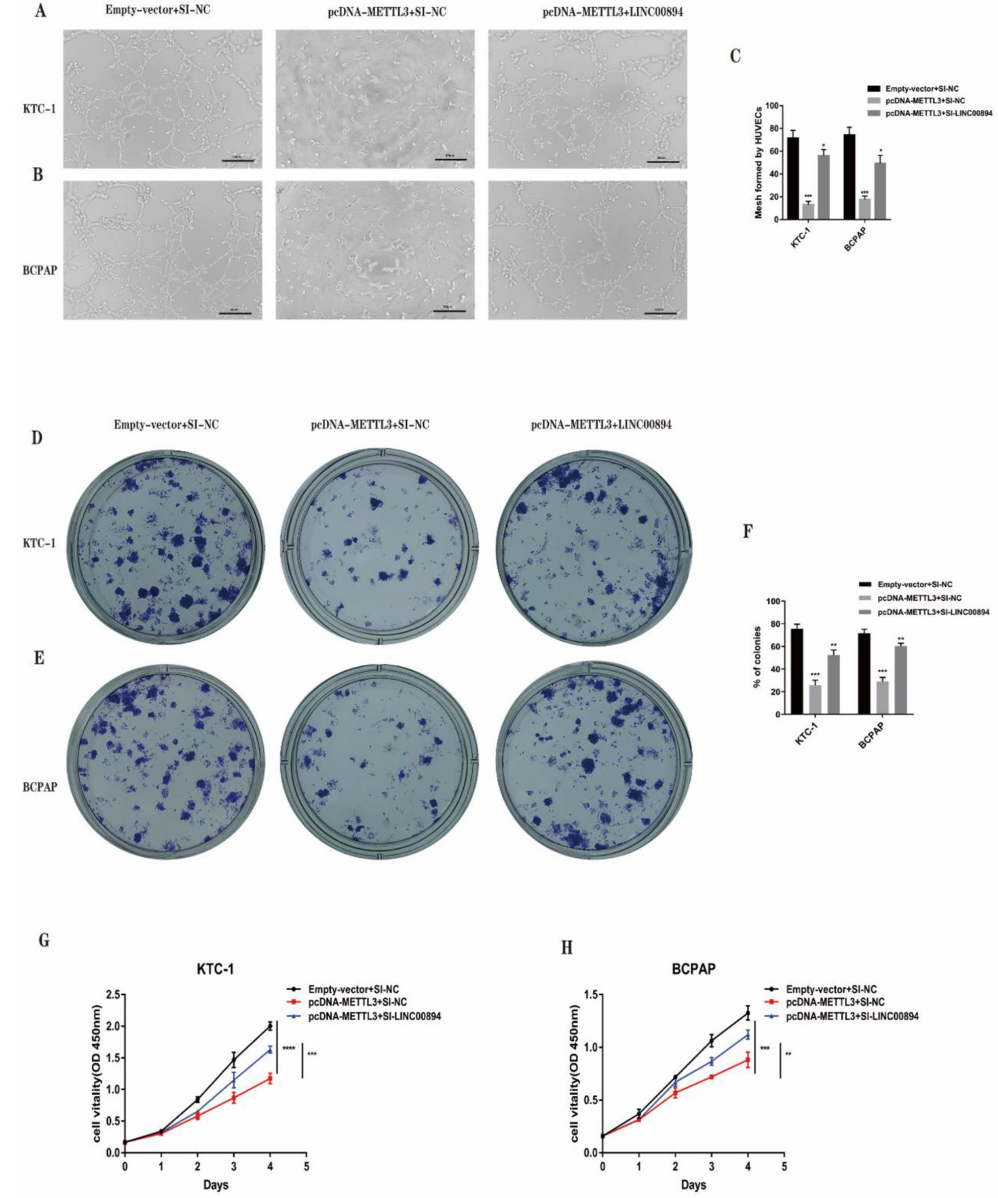
Reversal of the Tumour-Suppressive Effects of METTL3 by Silencing LINC00894. Functional rescue experiments, including the tube formation assay of HUVECs **(A-C)**, colony-forming assays **(D-F)**, and CCK-8 assays **(G-H)**, were performed. *P < 0.05, **P < 0.01, ***P < 0.001, ****P < 0.0001

To verify whether METTL3 contributes to the suppressive effects of PTC through LINC00894, we designed siRNA targeting LINC00894. Rescue experiments showed that by using METTL3-overexpressing PTC cells, HUVECs formed fewer meshes, while the inhibitory effects could be reversed by LINC00894 siRNA (Fig. 5A-C). According to the colony formation and CCK-8 assays, METTL3 overexpression also suppressed the proliferative abilities of PTC cells, and these effects could be reversed by LINC00894 siRNA transfection (Fig. 5D-H).

### LINC00894 inhibits PTC malignant phenotypes through the Hippo signalling pathway

**Fig. 6:**
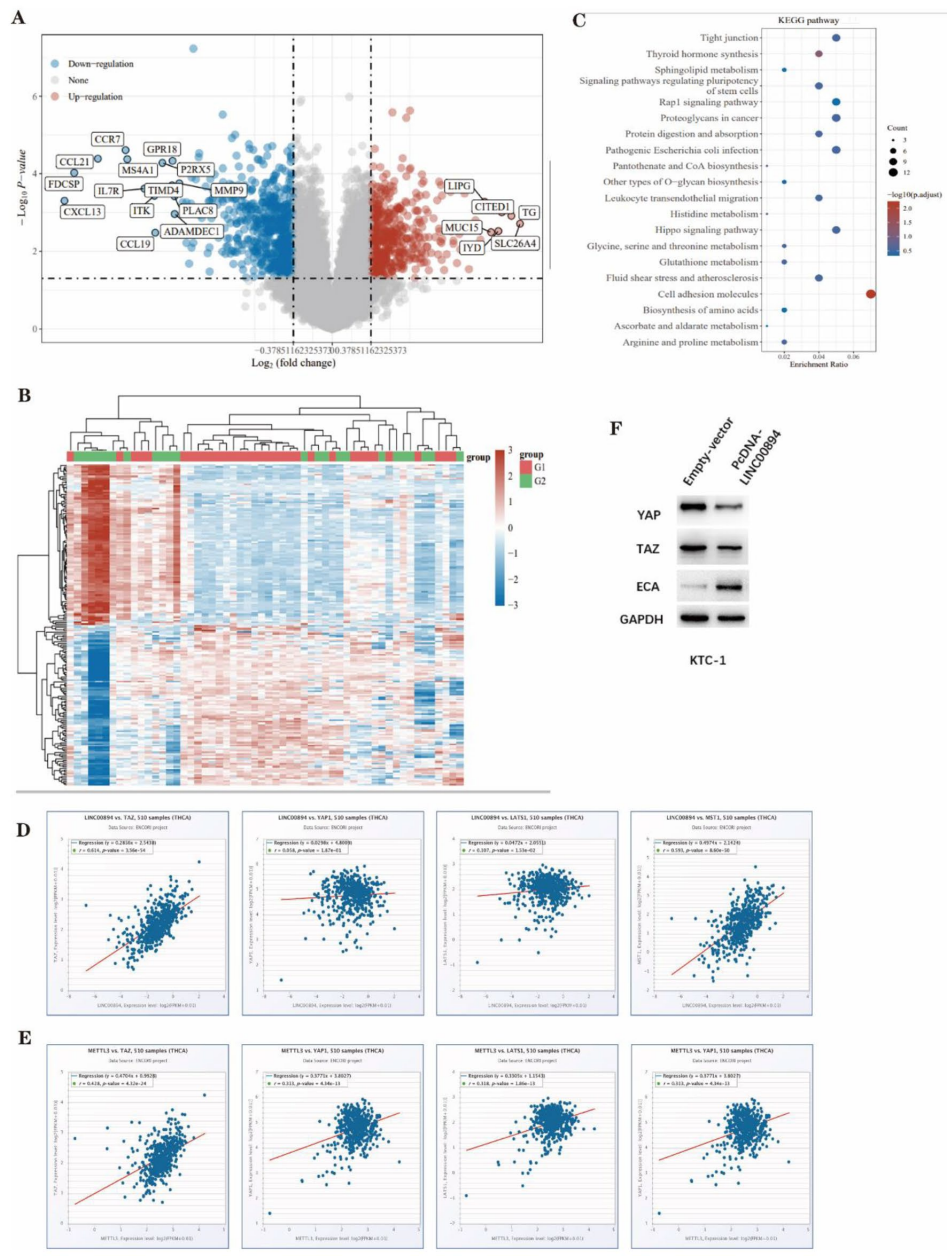
LINC00894 inhibits PTC malignant phenotypes through the Hippo signalling pathway. **A-B**. TCGA analysis was performed to identify differentially expressed genes in normal thyroid tissue and PTC tissue. **C.** KEGG pathway enrichment analysis. **D.** Analysis of the correlation between LINC00894 and TAZ, YAP, LATS1, and MST1 using the starBase database. **E.** Analysis of the correlation between METTL3 and TAZ, YAP, LATS1, and MST1 using the starBase database. **F.** Protein expression levels of Hippo signalling pathway-related proteins after pcDNA-LINC00894 transfection in KTC-1 cells according to Western blotting. *P < 0.05, **P < 0.01, ***P < 0.001, ****P < 0.0001

LncRNAs have been reported to be involved in tumours via various signalling pathways [35]. To explore the signalling pathway downstream of LINC00894, we first examined the differentially expressed genes (DEGs) in normal thyroid tissues and PTC tissues using the TCGA database (Fig. 6A-B). Subsequently, the RNA-seq data were analysed via the Kyoto Encyclopedia of Genes and Genomes (KEGG); the Hippo signalling pathway exhibited significant enrichment in the low LINC00894 expression group in comparison to the high LINC00894 expression group (Fig. 6C). Further analysis revealed that LINC00894 and METTL3 were significantly positive correlated with key components of the Hippo pathway (TAZ, YAP, LATS1, and MST1) (Fig. 6D-E). Then, Western blotting was used to examine LINC00894’s effect on related proteins in this pathway, revealing that after LINC00894 overexpression, YAP and TAZ protein expression decreased significantly, while the expression of ECA protein increased (Fig. 6F). Based on these findings, we deduced that LINC00894 may inhibit PTC malignant phenotypes via Hippo signalling.

## Discussion

Thyroid cancer has a high prevalence, and most cases are PTC, which is the most common endocrine malignancy [4]. Approximately 40% of adult PTC patients also have lymph node metastasis, which is correlated with higher recurrence rates and lower survival rates [36, 37]. Ultrasound, CT, and FDG-PET/CT have low sensitivity for the detection of lymph nodes positive for lateral and intercentral compartment tumours [38, 39]. There are only three main treatment options for differentiated thyroid cancer: surgical resection, TSH inhibition and radioactive iodine therapies; however, the lymph node metastasis rate within 20 years of unilateral thyroid lobotomy with DTC is 19%, and secondary surgery is the only option for patients at this time [40]. However, the heterogeneity among the histological and epigenetic characteristics of PTC is not well understood. Therefore, the identification or discovery of novel molecular markers in patients with PTC may improve the detection of metastatic lymph nodes and better guide treatment.

Multiple studies have revealed that lncRNAs are dysregulated in cancers with lymph node metastasis, and lncRNAs can exist in serum, plasma, or other body fluids in a stable form without being affected by endogenous RNA enzymes [41, 42], which makes lncRNAs a potential tumour marker for a variety of tumours [43]. LINC00894 contributes to the progression of various tumours, and the proliferative and invasive capabilities of kidney and lung cancer are closely related to its overexpression [16, 17]. In breast cancer, LINC00894 enhances breast cancer cell proliferation and invasion by competitively binding with miR-429 [18]. Nevertheless, studies on thyroid cancer have found that upregulation of LINC00894 can inhibit tumour cell activity [25]. LINC00894 has not yet been evaluated for its clinical significance and function in lymph node metastatic PTC. Herein, by using bioinformatics, LINC00894 expression was found to be significantly lower in PTC with lymph node metastasis than in PTC without lymph node metastasis, and the prognosis was worse when LINC00894 expression was lower. Functional tests indicated that LINC00894 overexpression significantly suppressed cell proliferation and lymphatic metastasis in vitro, whereas silencing LINC00894 induced the opposite effects. Based on these findings, we first elucidated the critical role of LINC00894 in the lymphatic metastasis of PTC.

Until now, no investigations have been conducted on the regulatory mechanism mediated by abnormal LINC00894 expression. Given the significant function of m6A modification in the regulation of gene expression in tumours, we further explored whether the downregulation of LINC00894 in PTC is related to m6A. By epigenetic transcriptomic analysis, we found that LINC00894 has a large number of methylation sites, and the m6A level is significantly positively correlated with METTL3 expression. It was confirmed that METTL3-induced m6A modification increased the stability of LINC00894 mRNA. The literature reports that METTL3 exhibits dual functionality as an oncogene and tumour suppressor [44]. METTL3 was reported to be downregulated in PTC and to suppress epithelial-mesenchymal transformation and Hedgehog signalling [29]. This is consistent with our findings. METTL3 is the main methyltransferase, and m6A readers, such as YTHDFs and IGF2BPs, recognize and act on m6A-modified mRNA transcripts [45, 46]. Further studies showed that overexpression of METTL3 and YTHDC2 significantly stabilized and promoted LINC00894 mRNA expression. The aforementioned findings suggested that METTL3 increases LINC00894 mRNA stability through the M6A-YTHDC2-dependent pathway. In recent years, targeting METTL3 to alleviate cancer progression has attracted much attention. Research has indicated that elvitegravir has the ability to suppress oesophageal cancer metastasis by increasing the proteasome degradation of the M6A methyltransferase METTL3 [47]. Our findings on the METTL3-LINC00894 axis, as well as its effect on PTC progression, will aid in the exploration of more effective treatment strategies for lymphatic metastasis of PTC.

Several signalling pathways have been demonstrated to participate in tumour inhibition or promotion by lncRNAs [48]. By KEGG enrichment analysis, the Hippo signalling pathway was indicated to be significantly enriched in PTC tissues with low LINC00894 expression. This signalling pathway regulates tissue growth, was first discovered in *Drosophila melanogaster* [49] and is highly conserved in humans. Studies in recent decades have revealed that the Hippo signalling pathway is dysfunctional in various malignancies, including liver [33], stomach [50], and lung cancers [51]. Dysregulation of the Hippo signalling pathway was also found in thyroid cancer [52]. In this study, it is innovatively proposed that LINC00894 regulates the Hippo signalling to affect PTC progression. This may be used as a potential therapeutic target for PTC treatment. However, the specific mechanism of this crosstalk remains to be determined, especially at the molecular level.

In conclusion, these results suggest that the METTL3-YTHDC2 axis regulates the stability of LINC00894 mRNA in an m6A-dependent manner and subsequently inhibits tumour malignancy through the Hippo signalling pathway.

## Data Availability

The datasets used and/or analysed during the current study are available from the corresponding author upon reasonable request.

## References

[1] Vaccarella S, Franceschi S, Bray F, Wild CP, Plummer M, Dal Maso L (2016). Worldwide thyroid- cancer epidemic? The increasing impact of overdiagnosis. N Engl J Med.

[2] Vaccarella S, Dal Maso L, Laversanne M, Bray F, Plummer M, Franceschi S (2015). The impact of diagnostic changes on the rise in thyroid cancer incidence: a population- based study in selected high- resource countries. Thyroid.

[3] Chen W, Zheng R, Baade PD (2016). Cancer statistics in China, 2015. CA Cancer J Clin.

[4] Riis MG, Juhl KS, Jens Meldgaard Bruun (2018). Concomitant sarcoidosis and papillary thyroid cancer with severe hypercalcaemia as the main symptom[J]. Bmj Case Reports.

[5] Qiu Y, Fei Y, Liu J (2019). Prevalence, risk factors and location of skip metastasis in papillary thyroid carcinoma: a systematic review and meta- analysis. Cancer Manag Res.

[6] Kluijfhout WP, Drake FT, Pasternak JD (2017). Incidental positive lymph nodes in patients with papillary thyroid cancer is independently associated with recurrent disease. J Surg Oncol.

[7] Harries LW (2012). Long non-coding RNAs and human disease. Biochem Soc Trans.

[8] Gupta RA, Shah N, Wang KC, Kim J, Horlings HM, Wong DJ, Tsai MC, Hung T, Argani P, Rinn JL, Wang Y, Brzoska P, Kong B, Li R, West RB, van de Vijver MJ, Sukumar S, Chang HY (2010). Long non-coding RNA HOTAIR reprograms chromatin state to promote cancer metastasis. Nature.

[9] Hung T, Wang Y, Lin MF, Koegel AK, Kotake Y, Grant GD, Horlings HM, Shah N, Umbricht C, Wang P, Wang Y, Kong B, Langerød A, Børresen-Dale AL, Kim SK, van de Vijver M, Sukumar S, Whitfield ML, Kellis M, Xiong Y, Wong DJ, Chang HY (2011). Extensive and coordinated transcription of noncoding RNAs within cell-cycle promoters. Nat Genet.

[10] Gong C, Maquat LE (2011). lncRNAs Transactivate STAU1-mediated mRNA decay by duplexing with 3’ UTRs via Alu elements. Nature.

[11] Qin Y, Hou Y, Liu S, Zhu P, Wan X, Zhao M, Peng M, Zeng H, Li Q, Jin T, Cui X, Liu M (2020). A Novel Long non-coding RNA lnc030 maintains breast Cancer stem cell stemness by stabilizing SQLE mRNA and increasing cholesterol synthesis. Adv Sci (Weinh).

[12] Sun D, Zhong J, Wei W, Liu L, Liu J, Lin X (2020). Long non-coding RNAs lnc-ANGPTL1-3:3 and lnc-GJA10-12:1 present as regulators of sentinel lymph node metastasis in breast cancer. Oncol Lett.

[13] Zheng S, Yang L, Zou Y, Liang JY, Liu P, Gao G, Yang A, Tang H, Xie X (2020). Long non-coding RNA HUMT hypomethylation promotes lymphangiogenesis and metastasis via activating FOXK1 transcription in triple-negative breast cancer. J Hematol Oncol.

[14] Huang C, Su X, Zhou DL, Xu BH, Liu Q, Zhang X, Tang T, Yang XH, Ye ZL, He CY (2022). A diagnostic and predictive lncRNA lnc-MPEG1-1 promotes the proliferation and metastasis of papillary thyroid cancer cells by occupying miR-766-5p. Mol Ther Nucleic Acids.

[15] Xiao J, Bing Z, Xiao G, Guan Y, Luan J (2020). Long non-coding (lnc)RNA PAPAS overexpression inhibits tumor growth in papillary thyroid carcinoma by downregulating lncRNA HOTTIP. Oncol Lett.

[16] Deng L, Wang P, Qu Z, Liu N (2021). The construction and analysis of ceRNA Network and immune infiltration in kidney renal clear cell carcinoma. Front Genet.

[17] Zhou Q, Li D, Zheng H, He Z, Qian F, Wu X, Yin Z, Bao PT, Jin M (2021). A novel lncRNA-miRNA-mRNA competing endogenous RNA regulatory network in lung adenocarcinoma and kidney renal papillary cell carcinoma. Thorac Cancer.

[18] Meng DF, Shao H, Feng CB (2021). LINC00894 enhances the progression of breast cancer by sponging miR-429 to regulate ZEB1 expression. Onco Targets Ther.

[19] Bokar JA, Rath-Shambaugh ME, Ludwiczak R, Narayan P, Rottman F (1994). Characterization and partial purification of mRNA N6-adenosine methyltransferase from HeLa cell nuclei. Internal mRNA methylation requires a multisubunit complex. J Biol Chem.

[20] Wang X, Feng J, Xue Y, Guan Z, Zhang D, Liu Z, Gong Z, Wang Q, Huang J, Tang C, Zou T, Yin P (2016). Structural basis of N(6)-adenosine methylation by the METTL3-METTL14 complex. Nature.

[21] Chen H, Pan Y, Zhou Q, Liang C, Wong CC, Zhou Y, Huang D, Liu W, Zhai J, Gou H, Su H, Zhang X, Xu H, Wang Y, Kang W, Kei Wu WK, Yu J (2022). METTL3 inhibits Antitumor Immunity by Targeting m6A-BHLHE41-CXCL1/CXCR2 Axis to promote Colorectal Cancer. Gastroenterology.

[22] Li Y, He X, Lu X, Gong Z, Li Q, Zhang L, Yang R, Wu C, Huang J, Ding J, He Y, Liu W, Chen C, Cao B, Zhou D, Shi Y, Chen J, Wang C, Zhang S, Zhang J, Ye J, You H (2022). METTL3 acetylation impedes cancer metastasis via fine-tuning its nuclear and cytosolic functions. Nat Commun.

[23] Liu H, Gu J, Huang Z, Han Z, Xin J, Yuan L, Du M, Chu H, Wang M, Zhang Z (2022). Fine particulate matter induces METTL3-mediated m6A modification of BIRC5 mRNA in bladder cancer. J Hazard Mater.

[24] Liu X, Zhang C, Wang X, Cui C, Cui H, Zhu B, Chen A, Zhang L, Xin J, Fu Q, Dionigi G, Sun H (2023). Long non-coding RNA MFSD4A-AS1 promotes lymphangiogenesis and lymphatic metastasis of papillary thyroid cancer. Endocr Relat Cancer.

[25] Chen B, Liu D, Chen R, Guo L, Ran J (2022). Elevated LINC00894 relieves the oncogenic properties of thyroid cancer cell by sponging let-7e-5p to promote TIA-1 expression. Discov Oncol.

[26] Wang J, Tan L, Jia B, Yu X, Yao R, OUYang N, Yu X, Cao X, Tong J, Chen T, Chen R, Li J (2021). Downregulation of m6A reader YTHDC2 promotes the Proliferation and Migration of malignant lung cells via CYLD/NF-κB pathway. Int J Biol Sci.

[27] Hu YP, Jin YP, Wu XS, Yang Y, Li YS, Li HF, Xiang SS, Song XL, Jiang L, Zhang YJ, Huang W, Chen SL, Liu FT, Chen C, Zhu Q, Chen HZ, Shao R, Liu YB (2019). LncRNA-HGBC stabilized by HuR promotes gallbladder cancer progression by regulating miR-502-3p/SET/AKT axis. Mol Cancer.

[28] Liu PI, Jiang YJ, Chang AC, Huang CL, Fong YC, Guo JH, Liu CL, Wang SW, Liu JF, Chang SL, Tang CH (2023). ANGPTL2 promotes VEGF-A synthesis in human lung cancer and facilitates lymphangiogenesis. Aging.

[29] Zhu Y, Peng X, Zhou Q, Tan L, Zhang C, Lin S, Long M (2022). METTL3-mediated m6A modification of STEAP2 mRNA inhibits papillary thyroid cancer progress by blocking the hedgehog signaling pathway and epithelial-to-mesenchymal transition. Cell Death Dis.

[30] Liu Q, Cui Y, Ding N, Zhou C (2022). Knockdown of circ_0003928 ameliorates high glucose-induced dysfunction of human tubular epithelial cells through the miR-506-3p/HDAC4 pathway in diabetic nephropathy. Eur J Med Res.

[31] Lei TX, He DJ, Cao J, Lv WG (2022). CircWDR26 regulates endometrial carcinoma progression via mir-212-3p-mediated typing genes MSH2. Eur J Med Res.

[32] Deng LJ, Deng WQ, Fan SR, Chen MF, Qi M, Lyu WY, Qi Q, Tiwari AK, Chen JX, Zhang DM, Chen ZS (2022). m6A modification: recent advances, anticancer targeted drug discovery and beyond. Mol Cancer.

[33] Wang J, Yu H, Dong W, Zhang C, Hu M, Ma W, Jiang X, Li H, Yang P, Xiang D (2023). N6-Methyladenosine-mediated Up-Regulation of FZD10 regulates Liver Cancer Stem cells’ properties and Lenvatinib Resistance through WNT/β-Catenin and Hippo Signaling Pathways. Gastroenterology.

[34] Ying Y, Ma X, Fang J, Chen S, Wang W, Li J, Xie H, Wu J, Xie B, Liu B, Wang X, Zheng X, Xie L (2021). EGR2-mediated regulation of m6A reader IGF2BP proteins drive RCC tumorigenesis and metastasis via enhancing S1PR3 mRNA stabilization. Cell Death Dis.

[35] Riquelme I, Pérez-Moreno P, Mora-Lagos B, Ili C, Brebi P, Roa JC (2023). Long non-coding RNAs (lncRNAs) as regulators of the PI3K/AKT/mTOR pathway in gastric carcinoma. Int J Mol Sci.

[36] Mazzaferri EL, Jhiang SM (1994). Long-term impact of initial Surgical and Medical Therapy on Papillary and follicular thyroid Cancer. Am J Med.

[37] Podnos YD, Smith D, Wagman LD, Ellenhorn JDI (2005). The implication of Lymph Node Metastasis on Survival in patients with Well-differentiated thyroid Cancer. Am Surg.

[38] Ahn JE, Lee JH, Yi JS, Shong YK, Hong SJ, Lee DH, Choi CG, Kim SJ (2008). Diagnostic accuracy of CT and Ultrasonography for evaluating metastatic cervical lymph nodes in patients with thyroid Cancer. World J Surg.

[39] Jeong H-S, Baek C-H, Son Y-I, Choi J-Y, Kim H-J, Ko Y-H, Chung J-H, Baek H-J (2006). Integrated 18F-FDG PET/CT for the initial evaluation of cervical node level of patients with papillary thyroid carcinoma: comparison with Ultrasound and contrast-enhanced CT. Clin Endocrinol.

[40] Haddad RI, Bischoff L, Ball D, Thyroid Carcinoma (2022). Version 2.2022, NCCN Clinical Practice guidelines in Oncology. J Natl Compr Canc Netw.

[41] Tinzl M, Marberger M, Horvath S, Chypre C (2004). DD3PCA3 RNA analysis in urine–a new perspective for detecting prostate cancer. Eur Urol.

[42] Isin M, Ozgur E, Cetin G, Erten N, Aktan M, Gezer U, Dalay N (2014). Investigation of circulating lncRNAs in B-cell neoplasms. Clin Chim Acta.

[43] Reis EM, Verjovski-Almeida S (2012). Perspectives of long non-coding RNAs in Cancer Diagnostics. Front Genet.

[44] Cai Y, Feng R, Lu T, Chen X, Zhou X, Wang X (2021). Novel insights into the m(6)A-RNA methyltransferase METTL3 in cancer. Biomark Res.

[45] Wang X, Lu Z, Gomez A, Hon GC, Yue Y, Han D (2014). N6-methyladenosine-dependent regulation of messenger RNA stability. Nature.

[46] Hsu PJ, Zhu Y, Ma H, Guo Y, Shi X, Liu Y (2017). Ythdc2 is an N6-methyladenosine binding protein that regulates mammalian spermatogenesis. Cell Res.

[47] Liao L, He Y, Li SJ, Zhang GG, Yu W, Yang J, Huang ZJ, Zheng CC, He QY, Li Y, Li B (2022). Anti-HIV drug Elvitegravir suppresses Cancer Metastasis via increased proteasomal degradation of m6A methyltransferase METTL3. Cancer Res.

[48] Tian Y, Chen ZH, Wu P, Zhang D, Ma Y, Liu XF, Wang X, Ding D, Cao XC, Yu Y. MIR497HG-Derived miR-195 and miR-497 mediate tamoxifen resistance via PI3K/AKT signaling in breast Cancer. Adv Sci (Weinh). 2023 Feb 23:e2204819. 10.1002/advs.202204819 PMC10131819 36815359

[49] Thompson BJ, Cohen SM (2006). The Hippo pathway regulates the bantam microRNA to control cell proliferation and apoptosis in Drosophila. Cell.

[50] Yan C, Yang H, Su P, Li X, Li Z, Wang D, Zang Y, Wang T, Liu Z, Bao Z, Dong S, Zhuang T, Zhu J, Ding Y (2022). OTUB1 suppresses Hippo signaling via modulating YAP protein in gastric cancer. Oncogene.

[51] Thirusangu P, Ray U, Sarkar Bhattacharya S, Oien DB, Jin L, Staub J, Kannan N, Molina JR, Shridhar V (2022). PFKFB3 regulates cancer stemness through the hippo pathway in small cell lung carcinoma. Oncogene.

[52] Song H, Qiu Z, Wang Y, Xi C, Zhang G, Sun Z, Luo Q, Shen C (2023). HIF-1α/YAP Signaling Rewrites Glucose/Iodine Metabolism Program to promote papillary thyroid Cancer progression. Int J Biol Sci.

